# Canine distemper virus isolated from a monkey efficiently replicates on Vero cells expressing non-human primate SLAM receptors but not human SLAM receptor

**DOI:** 10.1186/s12917-016-0757-x

**Published:** 2016-08-02

**Authors:** Na Feng, Yuxiu Liu, Jianzhong Wang, Weiwei Xu, Tiansong Li, Tiecheng Wang, Lei Wang, Yicong Yu, Hualei Wang, Yongkun Zhao, Songtao Yang, Yuwei Gao, Guixue Hu, Xianzhu Xia

**Affiliations:** 1 College of Animal Science and Technology, Jilin Agricultural University, Changchun, 130118 China; 2 Military Veterinary Research Institute of Academy of Military Medical Sciences, Key Laboratory of Jilin Province for Zoonosis Prevention and Control, Changchun, 130122 China; 3 National Research Center for Veterinary Medicine, Luoyang, Henan 471000 China; 4 College of Chemistry and Biology, Beihua University, Jilin, 132013 China; 5 Jiangsu Co-innovation Center for Prevention and Control of Important Animal Infectious Diseases and Zoonosis, Yangzhou, 225009 China; 6 Department of Animal Science and Veterinary Medicine, Henan Institute of Science and Technology, Xinxiang, 453003 China

**Keywords:** Canine distemper virus (CDV), Monkey, SLAM, H protein

## Abstract

**Background:**

In 2008, an outbreak of canine distemper virus (CDV) infection in monkeys was reported in China. We isolated CDV strain (subsequently named Monkey-BJ01-DV) from lung tissue obtained from a rhesus monkey that died in this outbreak. We evaluated the ability of this virus on Vero cells expressing SLAM receptors from dog, monkey and human origin, and analyzed the H gene of Monkey-BJ01-DV with other strains.

**Results:**

The Monkey-BJ01-DV isolate replicated to the highest titer on Vero cells expressing dog-origin SLAM (10^5.2±0.2^ TCID_50_/ml) and monkey-origin SLAM (10^5.4±0.1^ TCID_50_/ml), but achieved markedly lower titers on human-origin SLAM cells (10^3.3±0.3^ TCID_50_/ml). Phylogenetic analysis of the full-length H gene showed that Monkey-BJ01-DV was highly related to other CDV strains obtained during recent CDV epidemics among species of the Canidae family in China, and these Monkey strains CDV (Monkey-BJ01-DV, CYN07-dV, Monkey-KM-01) possessed a number of amino acid specific substitutions (E276V, Q392R, D435Y and I542F) compared to the H protein of CDV epidemic in other animals at the same period.

**Conclusions:**

Our results suggested that the monkey origin-CDV-H protein could possess specific substitutions to adapt to the new host. Monkey-BJ01-DV can efficiently use monkey- and dog-origin SLAM to infect and replicate in host cells, but further adaptation may be required for efficient replication in host cells expressing the human SLAM receptor.

## Background

Canine distemper virus (CDV) is a single-stranded, negative-sense, nonsegmented RNA virus of genus Morbillivirus within the family Paramyxoviridae. CDV is a highly contagious pathogen that can cause disease with high morbidity and mortality in immunologically naive hosts as a result of viral tropism for the cutaneous, respiratory, gastrointestinal, and central and peripheral nervous systems [1]. CDV has a broad host range and primarily affects animals belonging to the *Canidae* (e.g. dogs, wolves, and foxes) and *Mustelidae* (e.g. ferrets, badgers, and mink) families [2–4]. Previous studies had implicated CDV in the pathogenesis of Paget’s disease [5], and natural CDV infection of non-human primates has been reported [6–8]. In 2006, a CDV outbreak occurred in rhesus monkeys (Macaca mulatta) at a breeding farm in Guangxi province, China, with a morbidity rate (60 %) and a mortality rate (≈30 %), unexpectedly [6]. Two additional CD occurred in monkeys were reported in 2008. One occurred in rhesus monkeys at a laboratory animal center in Beijing with a reported 60 % (12/20) mortality [7], another occurred in Japan following the importation of cynomolgus monkeys (Macaca fascicularis) from China and was associated with a 10.6 % (46/432) mortality [8].

Host cell infection initiates with viral binding to receptor proteins on the surface of cells. The CDV viral envelope contains two integral glycoproteins, the hemagglutinin (H) protein and fusion (F) protein. The H protein mediates the binding of the virus to the cell membrane, and the F protein serves to fuse viral and host membranes, thereby enabling release of the viral contents into the cytoplasm [2]. The CDV-H glycoprotein mediates viral attachment through specific interactions with signaling lymphocyte activation molecule (SLAM) [9] or nectin-4 cellular receptors [10, 11]. SLAM is expressed on a subset of immune cells, while nectin-4 is expressed on epithelial cells of various organs. Although SLAM is a main receptor for morbilliviruses, each morbillivirus preferentially uses the SLAM of its host animals, as the specific residues within SLAM responsible for mediating interactions with the CDV-H protein can vary by species [12]. The specificity of the CDV-H protein for the SLAM receptor imposes species barriers and is partly responsible for restricting CDV host range [12, 13]. Nectin-4 is highly conserved among different animals [10]. Unlike the SLAM receptor, the characteristics of binding between nectin-4 and CDV were unknown. In this study, we evaluated the replication capacity of a CDV isolate obtained from a naturally infected monkey on Vero cells expressing dog, monkey, or human SLAM receptor proteins to better understand how CDV-H protein receptor specificity affects host range restriction.

## Results

### Surface expression of dog, monkey, and human SLAM on Vero cells

**Fig. 1:**
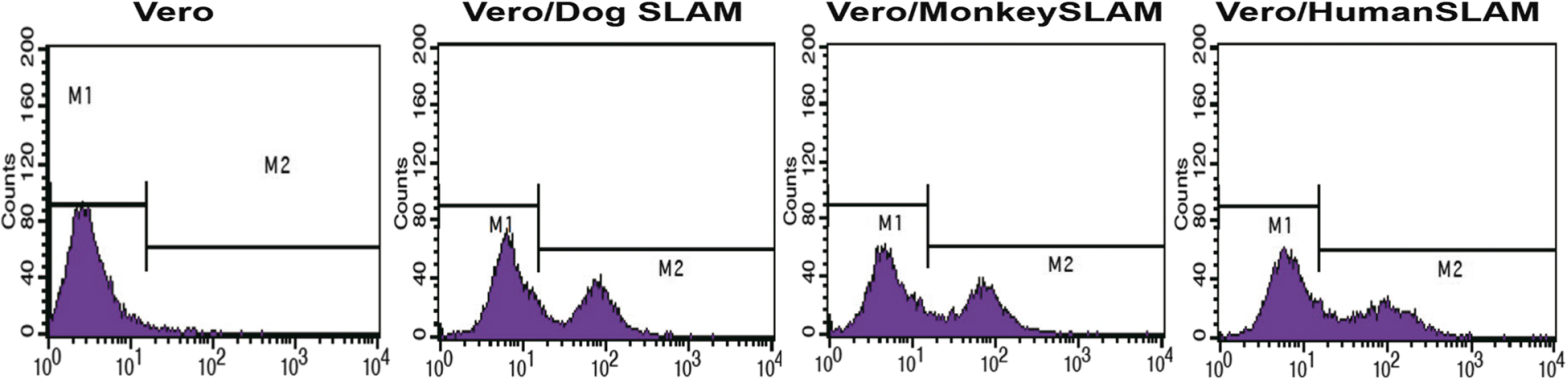
Stable expression of dog, monkey and human SLAM protein on Vero cells. Vero cells were transfected with expression plasmids to mediate expression of HA epitope-tagged SLAM proteins from dog, monkey, and human and selected with G418. The control Vero cells were transfected by empty vector. The resulting cell lines were named Vero/DogSLAM,Vero/MonkeySLAM and Vero/HumanSLAM. Anti-influenza virus HA epitope MAb was used to confirm surface expression of each version of the SLAM protein by flow cytometry

Vero cells were transfected with expression plasmids to express the dog, monkey, or human version of the SLAM protein. The expression plasmids included an HA epitope tag to allow confirmation of the surface expression of the SLAM protein. After cells stably transfected with G418 had been selected, SLAM expression was examined by flow cytometry using an HA tag-specific monoclonal antibody. The results showed that dog, monkey, and human SLAM was expressed equally on the surface of the resulting cell lines (46.5 %, 45.7 % and 45 %, respectively) but not on the empty vector-transfected Vero cells (Fig. 1). These cells were named Vero/DogSLAM, Vero/MonkeySLAM and Vero/HumanSLAM, respectively. For virus isolation study, these cells were passaged fewer than five times.

### Isolation of CDV from the lung tissue of a deceased rhesus monkey

**Fig. 2:**
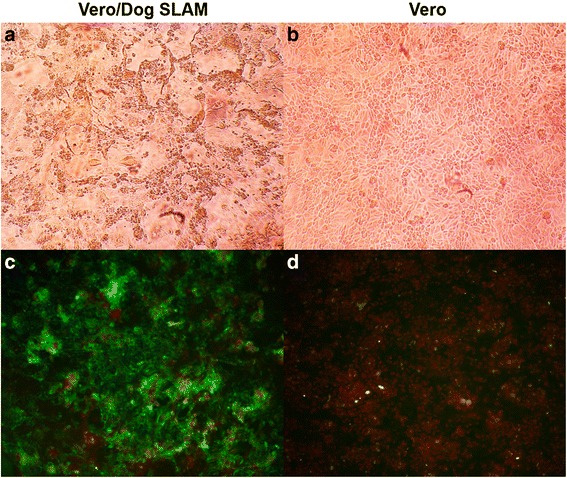
Infection of Vero cells expressing dog SLAM protein with CDV from a deceased rhesus monkey. Microscopic evaluation of Vero/DogSLAM cells (**a**) and Vero cells (**b**) following inoculation with Monkey-BJ01-DV at a MOI of 0.01. Inoculation of Vero/DogSLAM cells resulted in obvious CPE. Detection of CDV antigens in Vero cells expressing the dog SLAM protein (**c**) and untransfected Vero cells (**d**) 64 h following inoculation with Monkey-BJ01-CDV at a MOI of 0.01. Cells were fixed and probed with a mouse monoclonal anti-CDV nucleoprotein antibody. A FITC-conjugated goat anti-mouse secondary antibody was used for detection

We used the Vero/DogSLAM cells to isolate CDV from lung samples collected previously from a deceased rhesus monkey that had displayed signs of CD. Inoculation of the Vero/DogSLAM cells with supernatants from the homogenized tissue resulted in an obvious cytopathic effect (CPE), whereas no CPE was observed after four blind passages of the supernatants on the Vero cells (Fig. 2, upper panels). Nucleoprotein expression in the cells was analyzed by indirect immunofluorescence assay (IFA). The nucleoprotein antigen was detected in Vero/DogSLAM cells inoculated with homogenized tissue supernatants but was not detected the inoculated Vero cells (Fig. 2, lower panels). The viruses isolated from the inoculated Vero/DogSLAM cells were named Monkey-BJ01-DV.

### Phylogenetic analysis of the H protein of Monkey-BJ01-DV

We sequenced the full-length H protein of the Monkey-BJ01-DV (GenBank accession number KM923900) and performed a phylogenetic analysis of this strain and others deposited in GenBank (Table 1). The Monkey-BJ01-DV was highly similar to the other CYN07-dV monkey strains (AB687720; 99.8 %; 1823/1824 nucleotides) and Monkey-KM-01 (FJ405224; 99.0 %; 1807/1824 nucleotides), which were isolated from a cynomolgus monkey and from a rhesus monkey, respectively [6, 8]. These three monkey strains are each associated with CDV outbreaks among monkeys in China and belong to the Asia type I lineage. The CDV H protein is responsible for host cell attachment, is the most variable protein described for all members of the genus Morbillivirus and is an important determinant of the CDV host range [3]. We found that the H protein from these three strains of monkey-CDV possessed a number of amino acid specific substitutions compared to the H protein of CDV epidemic in other animals at the same period, as shown in Table 1: E276V, Q392R, D435Y and I542F. The glycine (G) residue at position 530 and the tyrosine (Y) residue at position 549, which correspond to the partial SLAM-receptor binding region, were conserved among all three monkey CDV isolates and among CDV isolates from members of *Canidae* and *Mustelidae* (Table 1).

**Table 1:** Amino acid differences between the hemagglutinin of Monkey-BJ01-DV and other CDV isolates

Amino acid sequence identity^a^	Sequence no.	24	276	365	392	435	530	542	549	597
98.4 %	JN896331 (PS-dog)	S	E	V	Q	D	G	I	Y	R
97.5 %	AB286946 (MD231-dog)	^b^	^b^	A	^b^	^b^	^b^	^b^	^b^	^b^
98.4 %	JQ732173 (LDH (06)-fox)	^b^	^b^	^b^	^b^	^b^	^b^	^b^	^b^	^b^
98.4 %	FJ848530 (BJ080514-dog)	^b^	^b^	^b^	^b^	^b^	^b^	^b^	^b^	^b^
98.2 %	EU325720 (fox-Hebei07)	^b^	^b^	^b^	^b^	^b^	^b^	^b^	^b^	^b^
98.0 %	EU325721 (fox-HLJ07)	^b^	^b^	^b^	^b^	^b^	^b^	^b^	^b^	^b^
97.4 %	EU325724 (mink-LN)	^b^	^b^	^b^	^b^	^b^	^b^	^b^	^b^	^b^
97.9 %	EU325728 (raccoon-JL07)	^b^	^b^	^b^	^b^	^b^	^b^	^b^	^b^	^b^
97.5 %	GQ332530 (dog-Wuhan)	^b^	^b^	^b^	^b^	^b^	^b^	^b^	^b^	^b^
97.3 %	CDV-TM-CC	^b^	^b^	^b^	^b^	^b^	^b^	^b^	^b^	^b^
-----	Monkey-BJ01-DV	F	V	A	R	Y	^b^	F	^b^	H
99.8 %	FJ405223 (monkey-BJ-01)	F	V	A	R	Y	^b^	F	^b^	^b^
99.8 %	AB687720 (mCNY07-dV)	F	V	A	R	Y	^b^	F	^b^	H
99.0 %	FJ405224 (monkey-KM-01)	^b^	V	A	R	Y	^b^	F	^b^	^b^

^a^Percent identity in hemagglutinin amino acid sequence when compared to Monkey-BJ01-DV

^b^Indicate the same residues as the ones of JN896331 (PS-dog)

### Replication of Monkey-BJ01-DV on Vero cells expressing SLAM from dog-, monkey-, or human- origin

**Fig. 3:**
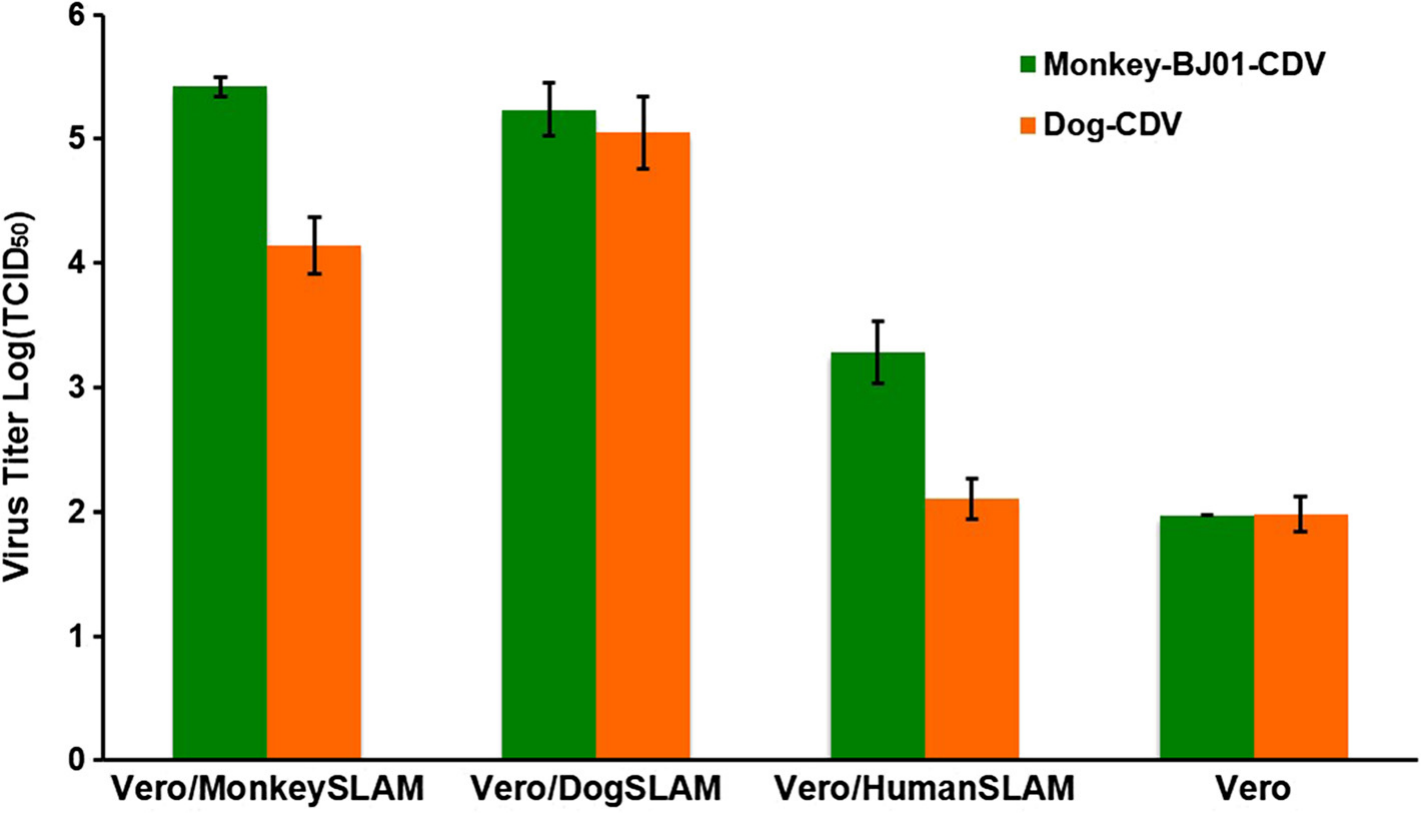
Replication of Monkey-BJ01-DV and CDV-TM-CC in Vero/SLAM cells. Cells were infected at a MOI of 0.01. Viral titers were measured 48 h post-infection

We next evaluated the replication ability of the Monkey-BJ01-DV in engineered Vero/SLAM cell lines. Monkey-BJ01-DV replicated to the highest titers on Vero/DogSLAM (10^5.2±0.2^ TCID_50_/ml) and Vero/MonkeySLAM (10^5.4±0.1^ TCID_50_/ml) at 48 h post-infection, whereas replication on the Vero/HumanSLAM cells was reduced approximately 100-fold (10^3.3±0.3^ TCID_50_/ml; Fig. 3). The dog-CDV isolate from a Tibetan mastiff replicated to high titers on the Vero/DogSLAM cells and displayed reduced titers on the Vero cells expressing SLAM from monkey- or human- origin. Our results indicate that both the monkey- and dog-CDV isolates can efficiently replicate on Vero cells expressing either the monkey- or dog-origin SLAM receptor, but replicate less efficiently on cells expressing the SLAM receptor of human- origin.

## Discussion

Morbilliviruses, including measles virus (MV), CDV, and rinderpest virus (RPV), are thought to have originated from a common ancestor several thousand years ago [14]. In general, however, each virus is able to naturally infect a relatively restricted number of host species. MV infection is limited to primates, CDV infection is limited to members of the *Canidae*, *Mustelidae* and *Procyonidae* families, and RPV infection is limited to ruminants [15]. Experimentally, monkeys are susceptible to MV infection and dogs to CDV infection [16]. Despite the apparent host range restriction of morbilliviruses, CDV has crossed species barriers, suggesting a potential ongoing expansion of host range. The first report of natural CDV infection of non-human primates was reported in 1989 among Japanese monkeys [15]. In 2006, a CDV epidemic affected a rhesus monkey (Macaca mulatta) breeding farm in China, inflicting high morbidity (60 %) and mortality (≈30 %) in young monkeys [6]. In 2008, Japan imported 432 cynomolgus monkeys (Macaca fascicularis) from China and 46 died from CDV while held in quarantine [8]. We isolated CDV from a deceased rhesus monkey (Monkey-BJ01-DV) following a 2008 outbreak in Beijing in which 12 of 20 rhesus monkeys died from CDV [7]. Collectively, these reports suggest significant changes in the epidemiology of CDV caused by an expanding CDV host range and/or viral virulence.

The complete genomes of three monkey-origin CDV isolates associated with outbreaks in China have been reported (Monkey-BJ01-DV (KF856711), CYN07-dV (AB687720) and MKY-KM-08 (HM852904) and are highly related to other epidemic CDV strains affecting species of the *Canidae* and *Mustelidae* families in China [6, 8] (Table 1). The H protein mediates viral attachment by binding to one or more cellular receptors [9, 11]. Several studies have speculated about the impact of specific amino acid substitutions within the H protein on interactions with the SLAM receptor. In vitro receptor-binding studies showed that amino acid residues 527, 528, 529, and 552 of the H protein are conserved among all morbilliviruses and are crucial for CDV-H to SLAM dependent fusion [4]. In particular, residues 530 and 549 both fall into receptor-binding domains located on propeller β-sheet 5 of the H protein [17]. Sequence analysis of the H gene of all three monkey CDV strains revealed a glycine (G) and a tyrosine (Y) at amino acid positions 530 and 549 of the partial SLAM-receptor binding region. G530 and Y549 are typically found in viral strains obtained from dogs in China, whereas other amino acid residues are present in CDV isolates obtained from wildlife [18, 19]. This suggests that the CDV isolated from monkeys was transmitted from domestic dogs rather than from other wildlife. In this study, we found that amino acid Y549 was conserved within CDV lineages of the isolates analyzed, regardless of host species. CDV transmission in wild carnivore and non-canid species may most often occur between individuals within a species, and may also be influenced by a range of additional factors such as population size and ranging patterns. The three monkey CDV strains possessed E276V, Q392R, D435Y and I542F substitutions, which are unique changes when compared to the other Asia type I lineage strains. In particularly, the I542F substitution falls with the SLAM-binding regions of the H protein. The H protein amino acid substitutions identified among monkey CDV isolates may help explain recent changes in CDV host range.

The Monkey-BJ01-DV can efficiently grow on the Vero cells expressing SLAM from dog- and monkey-origin, but not the cells expressing SLAM receptor of human- origin. Interestingly, while the amino acid sequence identity of dog and monkey SLAM is only 63.6 %, the Monkey-BJ01-DV strain can replicate on the Vero cells expressing SLAM receptor of dog-origin as efficiently as the Vero cells expressing monkey-origin SLAM. Factors other than the receptor binding, such as intracellular replication of the viruses may also be important for the establishment of infection. Further studies are required to understand the mechanisms by which CDV can cross species barriers.

## Conclusions

Canine distemper virus isolated from a deceased rhesus monkey efficiently replicates on the Vero cells expressing non-human primate SLAM receptors but not human SLAM receptor. The monkey origin-CDV-H protein could possess specific substitutions to adapt to the new host.

## Methods

### Plasmids and cell lines

Peripheral blood samples from a dog and rhesus monkey were collected from the Animal Hospital of Jilin University and the Animal Laboratory Center of the Academy of Military Medical Sciences, respectively. Human peripheral blood was kindly provided by the voluntary enrolled in this study. Peripheral blood mononuclear cells (PBMCs) of the dog, rhesus monkey and human were isolated from peripheral blood using dog and human lymphocyte separation media (TBD, Tianjin, China) according to the manufacturer protocols, respectively. Total RNA was extracted from the dog, rhesus monkey and human PBMCs after 2–4 h of stimulation with 2.5-3.0 mg of phytohemagglutinin (PHA) per milliliter and was used for reverse transcription with oligo (dT) primers. The presence and location of signal peptide cleavage sites in the amino acid sequences from the dog-SLAM (AF325357), rhesus monkey-SLAM (XM-001117605) and human-SLAM (NM-003037) were predicted by the Signal P 3.0 software. The SLAM gene of the dog, rhesus monkey and human without the signal peptide were encoded using various combinations of the primers designed on the basis of known SLAM sequences. The primer sets used were as follows: dogSLAM-F: 5′-GCCTCGAGACAGGTGAGAGCTTGATGAAT-3′ with the XhoI site underlined and dogSLAM-R: 5′-G CAGATCTTCAGCTCTCTGGGAACGTCAC-3′ with the BglII site underlined for dog; monkey/humanSLAM-F: 5′-GCCTCGAGGCAAGCTATGGAACAGGTGGG-3′; monkeySLAM-R: 5′-GCAGATCTTCAGCTCTCTGGAAGTGTCACACT-3′; and humanSLAM-R: 5′-TCAGATCTCTGGRARYGTCACRCT-3′), respectively. After checking against the NCBI reference sequence of dog SLAM, rhesus monkey SLAM and human SLAM, the cDNA encoding the DogSLAM, MonkeySLAM, and HumanSLAM proteins were inserted into the pCAGGS (Neo) vector possessing the immunoglobulin Igk leader sequence (GAG ACAGACACACTCCTGCTATGGGTACTGCTGCTCTGGGTTCCAGGTTCCACTGGTGAC) and the influenza virus hemagglutinin (HA) epitope (TATCCATATGATGTTCCAGATTATGCT) [20], generated pCAGDogSLAM, pCAGMonkeySLAM and pCAGHumanSLAM. Vero cells constitutively expressing dog SLAM (Vero/DogSLAM), rhesus monkey SLAM (Vero/MonkeySLAM) and human SLAM (Vero/HumanSLAM) were generated by transfecting Vero cells with pCAGDogSLAM, pCAGMonkeySLAM or pCAGHumanSLAM, respectively, and control Vero cells were transfected by empty vector. These cells were maintained in DMEM with 5 % FBS and 0.8 mg/ml Geneticin (G418; Invitrogen) in a humidified atmosphere at 37 °C and 5 % CO_2_. The above -described cells were stained with anti-influenza virus HA epitope MAb 12CA5 (Boehringer Mannheim), and then stained with fluorescein isothiocyanate (FITC)-conjugated goat anti-mouse IgG (Abcam, Cambridge science Park, UK). These stained cells were analyzed on a FACScan machine (Becton Dickinson) [2].

### Virus isolation

Tissue samples used for virus isolation were obtained from the lungs of a deceased rhesus monkey during the CDV epizootic in Beijing in 2008 [7]. Lung tissue was suspended in cold phosphate- buffered saline (PBS) with antibiotics and was grinded into a homogenate. Homogenized lung samples were centrifuged at 2500 rpm for 5 min; the supernatant was collected and centrifuged an additional 5 min at 5000 rpm. Supernatants were inoculated onto monolayers of Vero/DogSLAM cells, from which a CDV isolate was subsequently obtained and named Monkey-BJ01-DV. An additional CDV-TM-CC strain was isolated from a Tibetan mastiff in our laboratory [21]. Normal Vero cells and Vero/DogSLAM cells were plated in 24-well plates and infected with the Monkey-BJ01-DV. Productive CDV infection from the cultured cells was analyzed with a mouse monoclonal anti-CDV nucleoprotein antibody. A FITC-conjugated goat anti-mouse IgG was used as the secondary antibody, cells were analyzed with a fluorescence microscope (BX51FL; Olympus, Japan).

### RT-PCR and sequencing analysis of the Monkey-BJ01/Monkey-BJ01-DV

Total RNA was extracted from the lung of died monkey/Monkey-BJ01-DV by using TRIzol reagent (Molecular Research Center Inc., USA) according to the manufacturer instructions. Reverse transcription (RT) was carried out using the Superscript II reverse transcriptase (Invitrogen, USA) according to the standard protocol. The CDV-H gene was cloned using the Ex-Taq DNA polymerase (TaKaRa) with the following primers: CDV-HF: GCGAATTCATGCTCTCCTACCAAGACAAGGTG with the EcoRI site underlined and according to the CDV-HR: GGCCCTCGAGTCAAGGTTTTGAACGATTAC with XhoI site underlined. The PCR products were cloned into a pMD 18-T vector (TaKaRa) and were sequenced. At least five clones of each PCR products were analyzed to acquirie the accurate sequence. The sequence determined in this study had been registered at the GenBank under accession numbers FJ405223 and KM923900.

### Replication of CDV on Vero cells expressing the SLAM receptor from various animal species

Vero/DogSLAM, Vero/MonkeySLAM, and Vero/HumanSLAM cells and Vero cells (empty vector transfected cells) (1.8 × 10^5^ cells/well) were cultured in 24-well plates and infected with Monkey-BJ01-DV or CDV-TM-CC at a multiplicity of infection (MOI) of 0.01. The cells and supernatants were harvested 48 h post-infection and were stored at −80 °C until the virus titers were measured using the Vero/DogSLAM cells by the limiting dilution method and expressed as TCID_50_. Three independent experiments were performed to evaluate the CDV replication on the SLAM-expressing Vero cells.

## Abbreviations

CDV, canine distemper virus; CPE, cytopathic effect; F, fusion; FITC, fluorescein isothiocyanate; G, glycine; H, hemagglutinin; IFA, immunofluorescence staining; MOI, multiplicity of infection; MV, measles virus; PBMCs, peripheral blood mononuclear cells; PBS, phosphate-buffered saline; PHA, phytohemagglutinin; RPV, rinderpest virus; RT, reverse transcription; SLAM, signaling lymphocyte activation molecule; Y, tyrosine
